# Organic Sunscreens and Their Products of Degradation in Biotic and Abiotic Conditions—In Silico Studies of Drug-Likeness and Human Placental Transport

**DOI:** 10.3390/ijms252212373

**Published:** 2024-11-18

**Authors:** Anna W. Sobańska, Arkaprava Banerjee, Kunal Roy

**Affiliations:** 1 Department of Analytical Chemistry, Medical University of Lodz, Muszyńskiego 1, 90-151 Lodz, Poland; 2 Drug Theoretics and Cheminformatics Laboratory, Department of Pharmaceutical Technology, Jadavpur University, Kolkata 700032, India; arka.banerjee16@gmail.com

**Keywords:** placenta permeability, organic sunscreens, degradation products, QSAR, q-RASAR, ARKA

## Abstract

A total of 16 organic sunscreens and over 160 products of their degradation in biotic and abiotic conditions were investigated in the context of their safety during pregnancy. Drug-likeness and the ability of the studied compounds to be absorbed from the gastrointestinal tract and cross the human placenta were predicted in silico using the SwissADME software (for drug-likeness and oral absorption) and multiple linear regression and “ARKA” models (for placenta permeability expressed as fetus-to-mother blood concentration in the state of equilibrium), with the latter outperforming the MLR models. It was established that most of the studied compounds can be absorbed from the gastrointestinal tract. The drug-likeness of the studied compounds (expressed as a binary descriptor, ***Lipinski***) is closely related to their ability to cross the placenta (most likely by a passive diffusion mechanism). The organic sunscreens and their degradation products are likely to cross the placenta, except for very bulky and highly lipophilic 1,3,5-triazine derivatives; an avobenzone degradation product, 1,2-bis(4-tert-butylphenyl)ethane-1,2-dione; diethylamino hydroxybenzoyl hexyl benzoate; and dimerization products of sunscreens from the 4-methoxycinnamate group.

## 1. Introduction

Pregnant women are exposed to a variety of environmental pollutants—pesticides, drugs, substances of abuse or cosmetic raw materials that enter the body of a mother-to-be by different routes—orally (via contaminated food or water), through the skin or mucous membranes, or by inhalation. The placenta links the maternal and fetal blood circulations; molecules cross the placenta mainly via passive diffusion or (less frequently) using facilitated diffusion, placental transporters, phagocytosis or pinocytosis [1,2,3,4]. Apart from its protective functions, the main task of the placenta is to facilitate the transport of nutrients and oxygen to the fetus and remove wastes. However, the placenta must also be an “intelligent” barrier, permeable to desired substances and capable of stopping undesired ones [5]. Unfortunately, many unwanted compounds meet the criteria of good placenta transport [6,7] and are known or expected to cross the placenta barrier, so they are likely to affect the fetus. The placental permeability of compounds is vital in the context of their safety, but in vivo studies have ethical and technical limitations, and the results obtained with animal models [8,9,10] are not fully transferable to humans [1,9]. An increasing demand for ethically acceptable and effective methods to assess placenta permeability has led to the development of some ex vivo and in vitro models [3,11,12,13,14,15,16,17], including a novel trend of “organs on a chip”. Transport of compounds across the placenta has also been investigated in silico [5,18,19,20,21,22,23,24,25,26,27]. Placenta clearance index (***CI***) models were reported by Giaginis, Zhang, Chou, Hewitt, Guan, Wang and Gely [5,19,20,22,26,28]; fetal-to-maternal blood concentration ratios (***FM*s*)*** were predicted by Takaku, Eguchi, Wang and Lévêque [18,24,27,29]. The ex vivo parameter ***CI*** is usually expressed relative to a reference compound (antipyrine) to avoid inter-laboratory variances and differences arising from different placenta samples [29]. Both ***CI*** and ***FM*** are also used to classify compounds as easily (C)/not easily (NC) crossing the placenta in qualitative permeability studies (for ***CI***, the cut-off value is 0.8, and for ***FM*** it is 0.3 [23]). If both ***CI*** and ***FM*** values are available for a single compound, it happens in some cases that it is labeled as “C” based on one of the two parameters and as “NC” using another one. If such a situation takes place, classification based on the ***FM*** value prevails [23].

Human placenta permeability has been studied for different groups of compounds, in particular drugs and environmental pollutants such as pesticides, organophosphorus flame retardants, bisphenols, polycyclic aromatic hydrocarbons, polychlorinated dibenzo-p-dioxins and dibenzofurans, polychlorinated biphenyls, polybrominated ethers, perfluoroalkyl compounds, and chloronaphthalenes [18,20,24,25,28,30,31,32,33,34,35,36,37,38].

Organic sunscreens are expected to act on the surface of the skin or hair to protect them against damage caused by UV radiation; some sunscreens are also used to prevent photo-induced degradation of products—fabrics, cosmetic preparations, etc. Unfortunately, many compounds from this group meet the conditions of drug-likeness [39]. They are known to be absorbed transdermally [40], by the oral route or via the pulmonary tract [41]. Some organic sunscreens are found in mother’s milk, umbilical cord blood or placental tissues [33,42,43,44,45,46]. Organic sunscreens are not neutral to human and animal health; they are known endocrine disruptors and, generally speaking, can influence the development of offspring (especially male fetuses) [47,48,49,50,51] or are suspected neurotoxins [51,52]. The health risks associated with sunscreens’ environmental degradation products have attracted increasing attention [53], but to the best of our knowledge no systematic study of such compounds in the context of their ability to cross the placenta has been reported so far. In this study, the gap is filled with an in silico evaluation of the placenta permeability of organic sunscreens and their degradation products in biotic and abiotic conditions based on novel computational models. The studied sunscreens were selected to ensure that all the main chemical families involved in sun protection are represented and that as much information as possible is available on their environmental degradation pathways.

## 2. Results and Discussion

### 2.1. Human Placenta Permeability—Linear Regression Models

Compounds whose ability to cross the placenta has been measured experimentally are less abundant than, e.g., molecules whose experimental blood–brain barrier permeability data have been published [54]. So far, the largest libraries of compounds whose placenta permeability data are publicly available have been presented by Di Filippo (*n* = 248) [23] and Guan (*n* = 325) [28]. Both libraries contain binary qualitative permeability data compiled from several sources. Compounds are classified as crossing/not crossing the placenta based on their ***CI*** or ***FM*** values according to the criteria presented in Section 1, or their ability to cross the placenta has been established qualitatively. Sets of quantitative placenta permeability data are significantly smaller, as shown below:

1. Umbilical cord blood–maternal blood concentration ratios (***CM*s**) of 31 compounds [18];
2. Clearance indexes (***CI*s**) of 88 compounds [26];
3. Umbilical cord blood–maternal blood concentration ratios (***CM*s**) of 105 compounds [24];
4. Fetal–maternal blood concentration ratios (***FM*s**) of 55 compounds [27].

Datasets (a) to (d) have both advantages and disadvantages:

- Set (a) is very reliable because it contains data obtained according to the same methodology, but it is relatively small.
- Set (b) is sufficiently large for logical modeling, but, as stated above, ***FM*** or ***CM*** data are preferred over ***CI*** data.
- Set (c) is even larger than (a) and (b) and contains ***CM*** data, but it is a compilation of values obtained for specific compounds (organochlorine pesticides, hydroxylated polybrominated diphenyl ethers, polybrominated diphenyl ethers, polycyclic aromatic hydrocarbons, polybrominated biphenyl, hydroxylated polychlorinated biphenyls, polychlorinated biphenyls, polychlorinated dibenzo-p-dioxins, polychlorinated dibenzofurans, and per- and polyfluoroalkyl substances) whose physico-chemical properties are within relatively narrow ranges, not necessarily capturing the variability of the properties of the compounds investigated in this study.
- Set (d) proposed by Takaku contains compounds whose key features—lipophilicity (consensus log ***P*** = −1 to ca. 6), acid–base properties (neutral, weakly basic or weakly acidic), molecular size (maximum ***M***_w_ ca. 700) and polarity (***TPSA*** between 0 and 271)—are in closer agreement with those of the majority of studied solutes compared to set (c). It may therefore be expected that models based on set (d) will be more applicable in this study, obviating the need to extrapolate.

Based on the data provided by Takaku, some qualitative and quantitative models of human placenta permeability were proposed and applied to a large set of pesticides from different chemical families [25]. These models include Equation **(1)**, based on just two simple descriptors selected by forward stepwise multiple linear regression, and Equation **(2)**, which contains four descriptors calculated using Mordred software (version 1.2.0+galaxy0) and selected by forward stepwise multiple linear regression from the dataset reduced by the Partial Least Squares (PLS) method.

log ***FM*** = 0.14 (±0.12) − 0.0022 (±0.0003) ***M***_w_ + 0.0040 (±0.0008) ***TPSA***  **(1)**

(*n* = 40, R^2^ = 0.588, R^2^_adj_ = 0.568, F = 26.46, *p* < 0.01, Q^2^_LOO_ = 0.523, RMSECV = 0.254, RMSEP = 0.276, R^2^_ext_ = 0.468)

log ***FM*** = −0.038 (±0.162) − 0.0081 (±0.0025) ***ZMIC1*** − 0.011 (±0.002) ***EState_VSA8*** + 0.20 (±0.08) ***GATS7Z*** − 0.12 (±0.06) ***Lipinski***  **(2)**

(*n* = 40, R^2^ = 0.804, R^2^_adj._ = 0.781, F = 35.86, *p* < 0.01, Q^2^_LOO_ = 0.754, RMSECV = 0.184, RMSEP = 0.196, R^2^_ext_ = 0.721)

**Figure 1:**
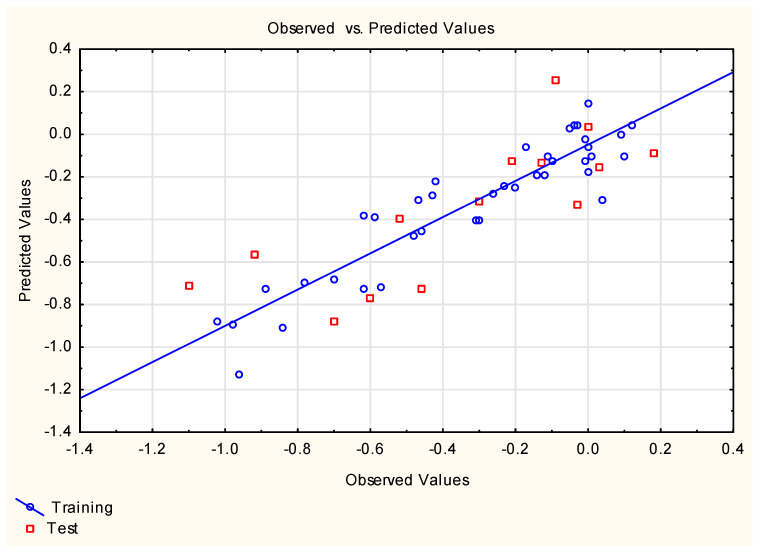
Equation **(3)**—predicted vs. experimental log ***FM*** values.

**Figure 2:**
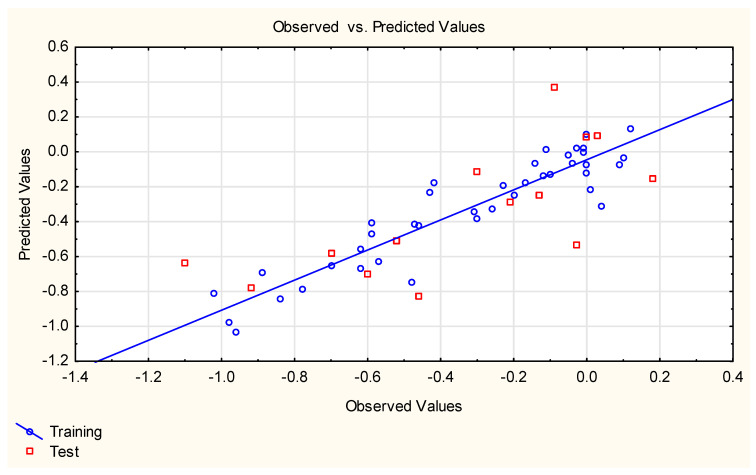
Equation **(4)**—predicted vs. experimental log ***FM*** values.

**Figure 3:**
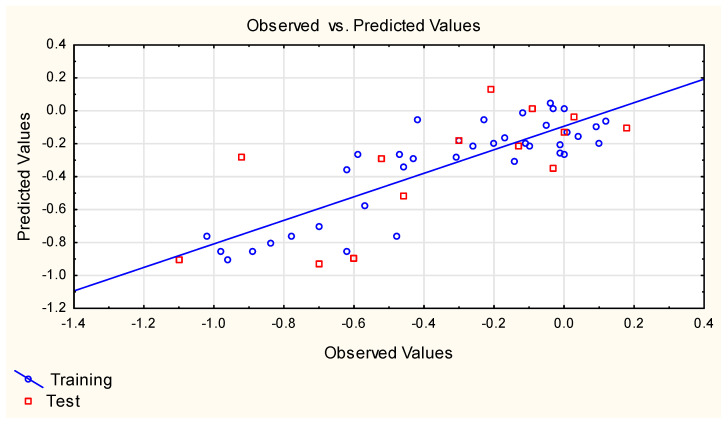
Equation **(5)**—predicted vs. experimental log ***FM*** values.

**Figure 4:**
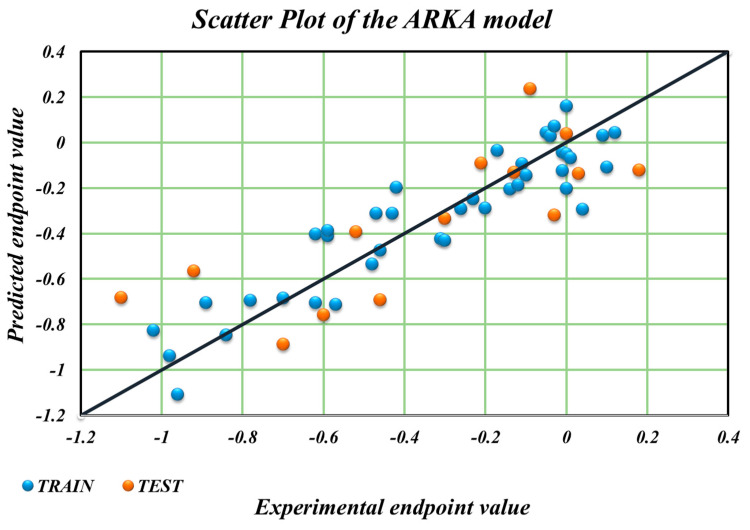
A scatter plot of the predicted vs. the experimental log ***FM*** values of the ARKA model (Equation **(6)**).

**Figure 5:**
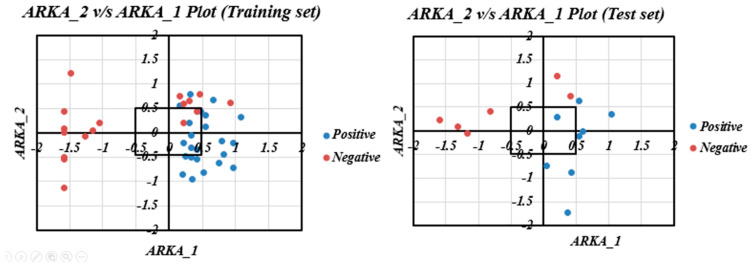
ARKA_2 vs. ARKA_1 plots of the training and test sets show the absence of activity cliffs; however, the presence of less confident data points (data points lying in the first and third quadrants) is evident.

Equation **(1)** is very simple and logical—it contains polar surface area (***TPSA***), a descriptor whose influence on compounds’ ability to cross different biological barriers has been reported by several authors [27,55,56], and ***M***_w_, which has been found to be negatively correlated with compounds’ placenta permeability [27]. However, the statistics of Equation **(1)** are sub-optimal, which prompted some other developments, e.g., Equations **(3)**–**(9)** (Figure 1, Figure 2, Figure 3, Figure 4 and Figure 5). Equations **(3)** and **(4)** account for 85 and 86% of total log ***FM*** variability, respectively; Equation **(5)** is based on two descriptors, of which the binary parameter ***Lipinski*** (generated using the Mordred software) is the primary one.

log ***FM*** = −0.68 (±0.05) + 0.53 (±0.06) ***Lipinski*** − 0.58 (±0.20) ***AATSC3s*** − 0.0058 (±0.0019) ***EState_VSA8*** − 9.78 (±4.11) ***JGI9*** + 0.45 (±0.17) ***AMID_N*** − 1.69 (±0.62) ***AATSC6se***  **(3)**

(*n* = 40, R^2^ = 0.851, R^2^_adj._ = 0.824, F = 31.49, *p* < 0.01, Q^2^_LOO_ = 0.776, RMSEP = 0.231, R^2^_ext_ = 0.617)

log ***FM*** = −0.51 (±0.14) + 0.29 (±0.09) ***Lipinski*** − 0.70 (±0.20) ***AATSC3s*** − 0.0013 (±0.0003) ***PNSA1*** − 0.0096 (±0.0018) ***EState_VSA8*** − 0.41 (±0.11) ***MATS7Z*** + 0.13 (±0.04) ***AATS3s***  **(4)**

(*n* = 40, R^2^ = 0.862, R^2^_adj._ = 0.837, F = 34.43, *p* < 0.01, Q^2^_LOO_ = 0.813, RMSEP = 0.270, R^2^_ext_ = 0.517)

log ***FM*** = −0.42 (±0.12) + 0.55 (±0.07) ***Lipinski*** − 0.12 (±0.03) ***iLOGP***  **(5)**

(*n* = 40, R^2^ = 0.715, R^2^_adj._ = 0.699, F = 46.34, *p* < 0.01, Q^2^_LOO_ = 0.673, RMSEP = 0.268, R^2^_ext_ = 0.513)

The independent variables involved in Equations **(3)**–**(5)** were selected using Partial Least Squares analysis, as described in Section 3.3 (stepwise reduction of an initial set of 1852 variables returned by Mordred and SwissADME platforms to just 84 variables), followed by forward stepwise regression. Equation **(5)** confirms the observation that placenta permeability is highly correlated with drug-likeness in the sense of Lipinski’s Ro5 [25]—and, as reported earlier, an important factor influencing the ability of a molecule to cross the placenta is lipophilicity [18]. Excessive lipophilicity of compounds impairs their placenta penetration measured in vivo. Compounds with ***M***_w_ values < 600 Da, which are rather hydrophobic and non-ionized, cross the placenta predominantly by passive diffusion [35]—in this case, since the lipid content in maternal blood is higher than that in fetal blood, more lipophilic molecules tend to remain in the maternal circulation [27]. A similar trend is observed for actively transported molecules—more hydrophilic compounds are more susceptible to transporter-mediated transfer across the placenta [57].

Log ***FM*** values were calculated for selected sunscreens and their degradation products according to Equations **(1)**–**(5)** (Supplementary Materials). The predicted log ***FM*** values were analyzed for the following: (i) sunscreens and degradation products known or expected to be poorly absorbed through biomembranes—diethylhexyl butamido triazone (DOBT), octocrylene (OCR), ethylhexyl triazone (ET), [2+2] cycloaddition products derived from ethylhexyl 4-methoxycinnamate (EHMC 18 to EHMC 22) and [2+2] cycloaddition products derived from isoamyl 4-methoxycinnamate (IMC 1 and IMC 2); and (ii) sunscreens/degradation products that have been proved to cross the placenta easily: butyl methoxydibenzoylmethane (avobenzone, BMDM), benzophenone-3 (BP3), benzophenone-4 (BP4), 4-hydroxybenzophenone (BP3 8) and 4-hydroxybenzoic acid (PABA 8) [42,51]. It was established that the calculated log ***FM*** values for the sunscreens/degradation products expected to cross the placenta easily were above the earlier mentioned cut-off value—and for the compounds whose transport through biological barriers is poor, the log ***FM*** values were below this threshold.

### 2.2. Results of the ARKA Analysis

The ARKA models that were developed using the descriptor combinations of Equations **(3)** and **(4)** are tabulated in Equations **(6)** and **(7)**, respectively.

(**6**) $$logFM=-0.3285+0.31401\times ARKA_{1}-0.26045\times ARKA_{2}$$

$$R^{2}=0.846,\;R_{adj}^{2}=0.838,\;Q_{LOO}^{2}=0.820,\;F=101.62,\;Q_{F1}^{2}=0.603,\;Q_{F2}^{2}=0.602,\;{RMSE}_{p}=0.233$$

(**7**) $$logFM=-0.3285+0.19721\times ARKA_{1}-0.28725\times ARKA_{2}$$

$$R^{2}=0.778,\;R_{adj}^{2}=0.766,\;Q_{LOO}^{2}=0.746,\;F=65,\;Q_{F1}^{2}=0.481,\;Q_{F2}^{2}=0.480,\;{RMSE}_{p}=0.266$$

From the different internal and external validation statistics, we can observe that Equation **(6)** is a much better model as compared to Equations **(3)** and **(4)** and Equation **(7)**. In fact, the robustness and the overall internal validation statistics of Equation **(6)** are better than those of all the developed models as well as the previously reported model [25]. Taking this model and its predictions, a scatter plot of the predicted vs. the experimental values was developed (Figure 4), and it can be observed that it shows minimal scattering. Additionally, the ARKA descriptors of Equation **(6)** were used to plot the ARKA_2 vs. ARKA_1 plot to identify potential activity/prediction cliffs, as shown in Figure 5. It was observed that no such activity cliffs were present in the training and test sets. However, there was the presence of many less confident data points. Please note that the ARKA_1 and ARKA_2 descriptors of Equation **(6)** are derived from the descriptors of Equation **(3)**, while those of Equation **(7)** are derived from the descriptors of Equation **(4)**.

If we compare the model statistics of the MLR QSAR models to the best ARKA model (Equation **(6)**), we find that the ARKA model not only generated higher cross-validation statistics (in terms of the leave-one-out Q^2^) but that the difference between the R^2^ and Q^2^_LOO_ values was also the lowest, justifying the high robustness of the ARKA model. Moreover, the ARKA model was developed using just two descriptors, whereas the other MLR QSAR models were developed using six descriptors. Statistical analysis also shows that the ARKA model has a much higher *F*-value compared to the MLR QSAR models. We also used the best ARKA model (Equation **(6)**) for the prediction of the true external set compounds (Supplementary Materials).

### 2.3. Results of the Read-Across and q-RASAR Models

Two different PLS q-RASAR models were developed using the two combinations of QSAR descriptors, as stated in Equations **(3)** and **(4)**. The developed PLS q-RASAR models derived from the descriptor spaces of Equations **(3)** and **(4)** are represented in Equations **(8)** and **(9)**, respectively, along with their different external, internal and cross-validated metrics that justify their robustness and predictivities. It is to be noted that both these PLS q-RASAR models supersede the quality not only of the corresponding QSAR models but also of the previously reported models (Equations **(1)** and **(2)**) [25].

(**8**) $$\begin{array}{l} logFM=-0.23463+0.33214\times Lipinski-0.00721\times EState_{VSA8}\\ \;\;\;\;\;\;\;+0.65269\times RA\;function-0.22397\times MaxPos \end{array}$$

$${LV=2,\;R}^{2}=0.800,\;R_{adj}^{2}=0.838,\;Q_{LOO}^{2}=0.757,\;F=74,\;Q_{F1}^{2}=0.742,\;Q_{F2}^{2}=0.741,\;{RMSE}_{p}=0.188$$

(**9**) $$\begin{array}{l} logFM=-0.34744-0.21423\times MATS7Z-0.00061\times PNSA1\\ \;\;\;\;\;\;\;+0.24892\times Lipinski-0.00714\times EState_{VSA8}\\ \;\;\;\;\;\;\;+0.25888\times g_{m} \end{array}$$

$${LV=2,\;R}^{2}=0.819,\;R_{adj}^{2}=0.809,\;Q_{LOO}^{2}=0.781,\;F=83.71,\;Q_{F1}^{2}=0.627,\;Q_{F2}^{2}=0.626,\;{RMSE}_{p}=0.226$$

The predictions derived from the best q-RASAR model (Equation **(8)**) for the external compounds are provided in the Supplementary Materials. The results from the non-statistical Read-Across approach were also quite good, the external validation metrics superseding those of the corresponding QSAR models as well as the previously reported models (as can be observed from the complete comparison table (Table 1)).

### 2.4. Drug-Likeness and Bioavailability

Organic sunscreens enter the environment in increasing quantities via different routes. They are washed off from the skin and hair during bathing or carelessly disposed of by manufacturers and consumers of cosmetic preparations. Sunscreens that circulate in the environment undergo degradation according to a variety of mechanisms, often leading to complex mixtures of products. In this study, a group of 16 organic sunscreens and over 160 stable degradation products obtained via different biotic and abiotic processes [58] were investigated in the context of their drug-likeness and human placenta permeability.

The drug-likeness (as defined by Lipinski Ro5 [59,60]) of some parent sunscreens was assessed earlier [39]—it was established that BP3, PABA, IMC, BMDM and MBC comply with the “Rule of 5” in all aspects; OMC, OCR, DHHB, ODP, HMS and OS exhibit not more than one violation of the “classical” Lipinski criteria. In this research, the selected sunscreens and their products of degradation in biotic and abiotic conditions were evaluated in the context of bioavailability and drug-likeness according to the Lipinski, Veber, Ghose, Palm, Egan and Muegge criteria [61,62], which assume the following properties of well-absorbed compounds:

- Lipinski (Mordred): ***M***_w_ ≤ 500, ***cLogP*** ≤ 5 (or ***MLOGP*** ≤ 4.15), #***HA*** ≤ 10, #***HD*** ≤ 5 [63];
- Lipinski (SwissADME);
- Veber: ***TPSA*** ≤ 140, #***FRB*** ≤ 10 [64];
- Ghose (Mordred): 160 ≤ ***M***_w_ ≤ 480, −0.4 ≤ ***WLOGP*** ≤ 5.6, 40 ≤ ***MR*** ≤ 130, 20 ≤ ***#Atoms*** ≤ 70 [65];
- Ghose (SwissADME);
- Palm: ***TPSA*** ≤ 140 [66];
- Egan: ***TPSA*** ≤ 131.6, ***WLOGP*** < 5.88 [67];
- Muegge: 200 ≤ ***M***_w_ ≤ 600, −2 ≤ ***XLOGP*** ≤ 5, #rings ≤ 7, #carbons > 4, #heteroatoms > 1, #***FRB*** ≤ 15, #***HA*** ≤ 10 [68].

Lipinski’s Ro5 and similar filters are very efficient in distinguishing between small molecules whose absorption is good or poor. However, they are just crude and simplistic rules that give falsely negative results for many macromolecules and actively transported drugs, e.g., registered protein kinase inhibitors [69]. Luckily, most of the molecules considered in this study fulfill Lipinski’s requirements; the possibility of other studied compounds being actively transported across the placenta is yet to be investigated.

**Figure 6:**
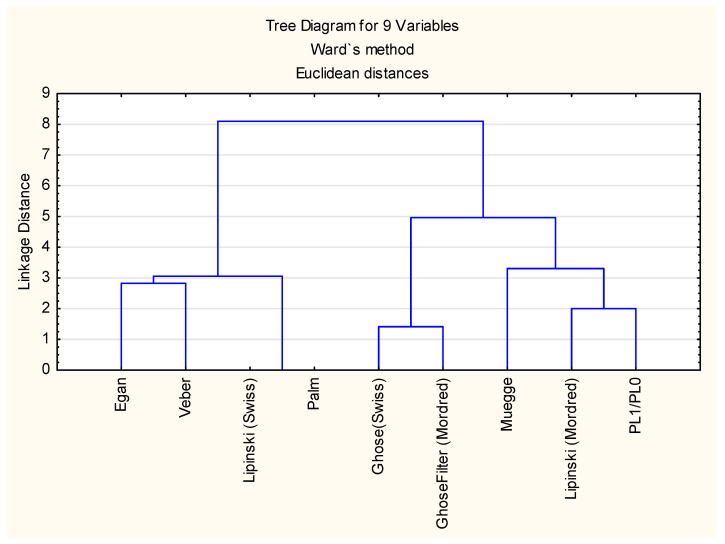
Comparison of the studied compounds’ ability to cross the placenta and their drug-likeness.

Lipinski and Ghose filters can be interpreted differently—according to some authors, no violation is accepted, and other researchers accept up to one violation of the rules; so, in some cases, different values of the same filter can be returned for the same compound [70,71]. The binary values of filters (i) to (viii) obtained for compounds ***1*** to ***54*** and the binary placenta permeability scores PL1/PL0 (with 1 representing likely high placenta permeability and 0 representing the opposite), defined earlier according to [25], were compared using Cluster Analysis (Figure 6). It was established that (irrespective of the amalgamation method and the distance measure) the filter that is particularly strongly linked to compounds’ placenta permeability is Lipinski, provided by Mordred (in the “strict” version, with no violations of the rules permitted).

According to Lipinski’s Ro5, the majority of the studied sunscreens and degradation products fulfill all the criteria of drug-likeness (Supplementary Materials) and are likely to cross biological barriers—the exceptions are very bulky and lipophilic sunscreens belonging to the family of 1,3,5-triazine derivatives (Et and DOBT—three violations of Ro5); one product of BMDM degradation (BMDM10—*tert*-butylbenzene); and compounds IMC1, IMC2, and EHMC20 to EHMC22—dimerization products of sunscreens from the p-methoxycinnamate group.

### 2.5. Placenta Permeability of the Studied Compounds vs. Structural Features

**Figure 7:**
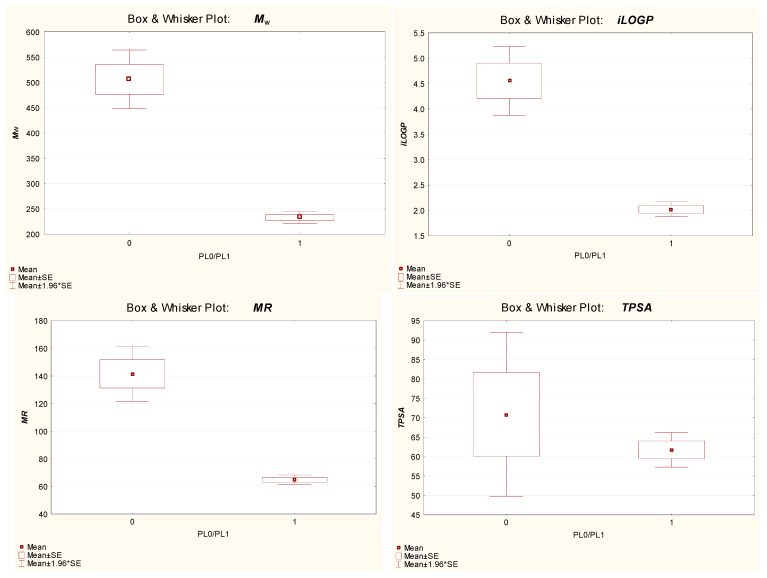
Comparison of key features of PL1 and PL0 compounds.

The molecules considered in this study (the reference set of 54 compounds, sunscreens and sunscreen degradation products) belonging to the PL1 and PL0 groups (good and poor placenta penetrators, respectively) differ significantly in terms of lipophilicity (***iLOGP***), size (***M***_w_) and polarizability (***MR***), but not so much in terms of polarity (***TPSA***) (Figure 7). Considering the drug-likeness of parent sunscreens and their known or predicted ability to cross the placenta and/or the properties of sunscreen degradation products, one may assume that almost all the studied organic sunscreens are, potentially, a health issue during pregnancy, with the possible exceptions of DHHB, ET and DOBT.

The qualitative approach to the placenta permeability of the studied sunscreens and degradation products revealed that the molecules with the highest fetus-to-mother concentration ratios (log ***FM*s**) were the derivatives of PABA and Et-PABA, which is a positive development, considering that PABA is banned or avoided for use in cosmetics in many countries and that Et-PABA is a common impurity in preparations containing PABA and PABA esters as sunscreens [72]. Other compounds with particularly high ***FM*** ratios are products of the degradation of UV filters, which are, informally, not recommended in pregnancy and have already been banned in some coastal regions (e.g., Hawaii [73]) due to environmental issues (BP3, BMDM and EHMC). Sunscreens such as BP3, BMDM, EHMC and EHDP cross the placenta barrier with ease, and each of them is a source of ca. 20–30 degradation products of suspected good placenta permeability, whose health impacts, once they have reached the fetal circulation, are largely unknown.

## 3. Materials and Methods

### 3.1. Compounds

Experimental log ***FM*** values of compounds ***1*** to ***54*** were used to generate the quantitative models of placenta permeability described in Section 2. The data were taken from [29] and are listed in the Supplementary Materials. These compounds were divided into two sets: a training set (compounds ***1*** to ***40***), which was used for the construction of mathematical models, and a test set (compounds ***41*** to ***54***), which was used to evaluate the model’s performance on unseen data.

### 3.2. Calculated Descriptors

Molecular weight (***M****_w_*), heavy atom count (***#HvAt***), aromatic heavy atom count (***#ArHvAt***), fraction of sp^3^ carbons (***F***_Csp3_), freely rotatable bond count (#***FRB***), hydrogen donor count (#***HD***), hydrogen acceptor count (#***HA***), molar refractivity (***MR***), octanol–water partition coefficient (***iLOGP***) and topological polar surface area (***TPSA***) were calculated using the SwissADME platform (http://www.swissadme.ch, accessed on 1 October 2024) [71]. Mordred 2D and 3D descriptors (including ***Lipinski***) [70] were calculated using the OCHEM platform (https://ochem.eu, accessed on 1 October 2024), with Baloon optimization (3D) [74]. The calculated molecular descriptors for compounds ***1*** to ***54*** and 181 sunscreens and their degradation products are available in the Supplementary Materials.

### 3.3. Multiple Linear Regression

Multiple linear regression (MLR) models were generated using Statistica v. 13.3 by StatSoft Polska, Kraków, Poland, using the stepwise forward regression mode. Partial Least Squares (PLS) selection of independent variables suitable for MLR analysis from a pool of descriptors listed in Section 3.2 was performed using Statistica v. 13.3 and the NIPALS algorithm with auto-scaling for a training set of 40 compounds. Variables with zero variation or those that were very strongly co-linear were discarded automatically. The remaining variables were processed using the Partial Least Squares (PLS) methodology—those with Variable Importance in the Projection (VIP) values <1 were rejected, and the procedure was repeated [75].

Regression models of log ***FM*** were evaluated by internal validation, using the following parameters: cross-validated explained variance in prediction (leave-one-out cross-validation, Q^2^_LOO_); determination coefficient of the training set (R^2^); root mean square error of Leave-Many-Out (LMO) cross-validation (8-fold; RMSECV). An external test set of compounds (***41*** to ***54***) was used to evaluate the model performance on unseen data by root mean square error of external prediction (RMSEP) and determination coefficient of external prediction (R^2^_ext_) [76,77].

### 3.4. Development of the ARKA Model and Analysis of Activity Cliffs

One of the recent developments in cheminformatics involves the use of supervised dimensionality reduction techniques like Arithmetic Residuals in *K*-groups Analysis (ARKA), which can not only be efficiently used to detect activity and prediction cliffs but also to develop predictive models in some cases [78]. In the present study, we computed ARKA descriptors using the modeling descriptors of Equations **(3)** and **(4)**. The computed ARKA descriptors were then used to develop simple MLR models. Additionally, identification of activity cliffs was performed based on the ARKA descriptors computed from Equation **(3)**.

### 3.5. Generation of Read-Across Predictions and Development of q-RASAR Models

Read-Across is a powerful technique used in predictive toxicology, especially in cases where there are limited numbers of experimental data points [79,80], like in the present study. We performed Read-Across predictions using the tool Read-Across-v4.2.2 (available from https://sites.google.com/jadavpuruniversity.in/dtc-lab-software/home, accessed on 1 October 2024). This tool performs Read-Across predictions based on three different similarity considerations, namely, Euclidean distance-based similarity, Gaussian kernel-based similarity and Laplacian kernel-based similarity. Computing Read-Across predictions involves certain hyperparameters like σ, γ and the number of close source compounds that need to be optimized prior to the generation of predictions. Adhering to the basic theories of machine learning, we divided the training set into sub-training and validation sets and generated a grid search of Read-Across predictions for the validation set using the tool Auto_RA_Optimizer-v1.0 (available from https://sites.google.com/jadavpuruniversity.in/dtc-lab-software/home, accessed on 1 October 2024). The combination of hyperparameters that provided the best prediction for the validation set was used to generate the Read-Across predictions of the original test set, taking the original training set as the source set. The prediction qualities were evaluated using standard external validation metrics like Q^2^_F1_, Q^2^_F2_ and RMSEP.

For the development of the q-RASAR models [81,82,83,84], the optimized hyperparameter setting from the Read-Across was used to compute the similarity and error-based RASAR descriptors using the tool RASAR-Desc-Calc-v3.0.3 (available from https://sites.google.com/jadavpuruniversity.in/dtc-lab-software/home, accessed on 1 October 2024). After the computation of the RASAR descriptors, the originally selected QSAR descriptors and the computed RASAR descriptor matrices were fused to generate a complete descriptor pool [85,86]. This combined pool was subjected to feature selection using the Best Subset Selection tool (available from https://teqip.jdvu.ac.in/QSAR_Tools/, accessed on 1 October 2024), which uses a grid search algorithm to generate MLR models using different combinations of descriptors. The best MLR q-RASAR model was selected based on the cross-validated Q^2^ statistics, and further subjected to stringent internal and external validation. Finally, a Partial Least Squares (PLS) model was developed to obviate inter-correlation among the modeling descriptors, if any [87]. The above-mentioned procedure mentioned in this section was performed twice, using the two different QSAR models with two different modeling descriptors.

## 4. Conclusions

A total of 16 organic sunscreens and over 160 products of their degradation in biotic and abiotic conditions were investigated in the context of their safety during pregnancy using quantitative models of placenta permeability developed based on a group of compounds whose experimental fetus-to-mother blood concentration ratio (log ***FM***_vivo_) values are known.

The drug-likeness of the studied compounds was predicted in silico using different filters, Lipinski’s Ro5 having the closest relationship to the ability to cross the placenta. It was established that the majority of the studied compounds can enter a human body by the oral route, e.g., via contaminated food or water.

The ability of the studied compounds to cross the placenta barrier (log ***FM***) was predicted qualitatively using different models from multiple linear regression, Read-Across, quantitative Read-Across Structure–Activity Relationship (q-RASAR) and the novel Arithmetic Residuals in K-groups Analysis (ARKA) families, the ARKA and MLR q-RASAR models outperforming the MLR QSAR models in terms of their predictive ability. It was found that the majority of the studied organic sunscreens and their degradation products have the potential to cross the placenta, with the exception of the very bulky and highly lipophilic 1,3,5-triazine derivatives; a BMDM degradation product, 1,2-bis(4-tert-butylphenyl)ethane-1,2-dione; diethylamino hydroxybenzoyl hexyl benzoate; and dimerization products of sunscreens from the 4-methoxycinnamate group.

Organic sunscreens and the products of their degradation in aquatic environments in biotic and abiotic conditions should be monitored since they are likely to affect the well-being of humans at a prenatal stage of their development. However, some knowledge gaps remain to be filled—the possibility of transport of sunscreens/degradation products by transport mechanisms other than passive diffusion is, so far, unexplored and some studies in this area are underway; future studies on the long-term environmental effects of the studied degradation products are strongly recommended. Additionally, the placenta is not just a physical barrier but also an enzymatic one aimed at the deactivation of harmful xenobiotics. Further research is in progress to establish whether the studied compounds are likely to impair this protective function of the placenta.

## Figures and Tables

**Table 1:** Comparison of the statistical quality of the main models (QSAR, q-RASAR, ARKA and Read-Across) *.

Features from	Model	nDesc	LVs	Model Type	Training Set Statistics				Test Set Statistics		
					$R^{2}$	$R_{adj}^{2}$	$Q_{LOO}^{2}$	F-Value	$Q_{F1}^{2}$	$Q_{F2}^{2}$	RMSEP
**Equation (3)**	QSAR	**6**	**-**	MLR	0.851	0.824	0.776	31.5	0.608	0.607	0.231
	ARKA	2	-	MLR	0.846	**0.838**	**0.820**	**101.6**	0.603	0.602	0.233
	Read-Across	6	-	-	-	-	-	-	0.729	0.729	0.192
	q-RASAR	4	-	MLR	0.813	0.792	0.765	38.077	0.726	0.725	0.193
	q-RASAR	4	2	PLS	0.800	0.789	0.757	74.000	**0.742**	**0.741**	**0.188**
**Equation (4)**	QSAR	6	-	MLR	0.862	0.837	0.813	34.427	0.464	0.463	0.270
	ARKA	2	-	MLR	0.778	0.766	0.746	65.000	0.481	0.480	0.266
	Read-Across	6	-	-	-	-	-	-	0.705	0.704	0.200
	q-RASAR	5	-	MLR	0.823	0.797	0.775	31.697	0.604	0.603	0.232
	q-RASAR	5	2	PLS	0.819	0.809	0.781	83.710	0.627	0.626	0.226

* The best statistics and the best models are highlighted in **BOLD**. nDesc = number of descriptors, LVs = latent variables, RMSEP = root mean square error of prediction.

## Data Availability

Data generated in this study can be found in the manuscript.

## Supplementary Materials

The following supporting information can be downloaded at: https://www.mdpi.com/article/10.3390/ijms252212373/s1.
